# Preparation and Antimicrobial Activity of Chitosan and Its Derivatives: A Concise Review

**DOI:** 10.3390/molecules26123694

**Published:** 2021-06-17

**Authors:** Luminita Georgeta Confederat, Cristina Gabriela Tuchilus, Maria Dragan, Mousa Sha’at, Oana Maria Dragostin

**Affiliations:** 1 Department of Microbiology, Faculty of Medicine, “Grigore T. Popa” University of Medicine and Pharmacy, 16 Universitatii Street, 700115 Iasi, Romania; 2 Department of Drug Industry and Pharmaceutical Biotechnologies, Faculty of Pharmacy, “Grigore T. Popa” University of Medicine and Pharmacy, 16 Universitatii Street, 700115 Iasi, Romania; maria.wolszleger@umfiasi.ro; 3 Department Pharmaceutical Technology, Faculty of Pharmacy, “Grigore T. Popa” University of Medicine and Pharmacy, 16 Universitatii Street, 700115 Iasi, Romania; mousa-shaat@umfiasi.ro; 4 Research Centre in the Medical-Pharmaceutical Field, Faculty of Medicine and Pharmacy, “Dunarea de Jos” University of Galati, 47 Domneasca Street, 800008 Galati, Romania; oana.dragostin@ugal.ro

**Keywords:** chitosan, derivatives, antimicrobial activity

## Abstract

Despite the advantages presented by synthetic polymers such as strength and durability, the lack of biodegradability associated with the persistence in the environment for a long time turned the attention of researchers to natural polymers. Being biodegradable, biopolymers proved to be extremely beneficial to the environment. At present, they represent an important class of materials with applications in all economic sectors, but also in medicine. They find applications as absorbers, cosmetics, controlled drug delivery, tissue engineering, etc. Chitosan is one of the natural polymers which raised a strong interest for researchers due to some exceptional properties such as biodegradability, biocompatibility, nontoxicity, non-antigenicity, low-cost and numerous pharmacological properties as antimicrobial, antitumor, antioxidant, antidiabetic, immunoenhancing. In addition to this, the free amino and hydroxyl groups make it susceptible to a series of structural modulations, obtaining some derivatives with different biomedical applications. This review approaches the physico-chemical and pharmacological properties of chitosan and its derivatives, focusing on the antimicrobial potential including mechanism of action, factors that influence the antimicrobial activity and the activity against resistant strains, topics of great interest in the context of the concern raised by the available therapeutic options for infections, especially with resistant strains.

## 1. Introduction

In recent years, the interest in using natural and synthetic polymers for biomedical and pharmaceutical applications significantly increased. In this regard, there have been investigated a wide variety of biodegradable and nonbiodegradable polymers [1]. The most explored polymers were chitosan, hyaluronic acid, dextran, alginate, starch, cellulose, gelatin as natural polymers and polylactic acid, polylactic-coglycolic acid, poly-caprolactone, acrylic polymers, polyethylene glycol as synthetic polymers [2,3,4]. Unfortunately, most plastics are not biodegradable and come from nonrenewable sources. On the other hand, the properties underpinning their multiple applications, such as strength and durability, are also responsible for the lack of biodegradability and persistence in the environment for a long time [5].

Given the problems of toxicity and difficulty in removal raised by nonbiodegradable polymers [1], natural polymers are preferred for biomedical applications, fulfilling the most important criteria to be used for this purpose as biocompatibility, biodegradability, low or nontoxicity and low immunogenicity. Moreover, natural polymers are readily available and cost-effective, their exploitation becoming of great interest [1,6,7]. Despite the many advantages, natural polymers have the disadvantage of susceptibility to microbial contamination. 

Natural compounds have constantly been a source of inspiration in the attempt to develop new drugs, due to their unique and complex chemical composition, responsible for a large variety of biological effects [8]. In this regard, some natural polymers proved a promising pharmacological potential, as well as suitability as drug delivery systems, these two research directions being of great interest at this moment, focusing a large amount of work [9,10,11]. 

The need to develop new effective antimicrobial agents is imperatively increased as long as the treatment of the infectious diseases represents, at this moment, a serious concern and a continuous challenge [12]. This concern is related to factors such as the limited access to appropriate antimicrobial agents, the unfavorable safety profile of some classes of antibiotics and, not at least, the rapid development of antibiotic resistance phenomenon [13]. The emergence of the antimicrobial resistance among microbial strains and the rapid spread of the resistant microorganism, coupled with the slow rate of developing new antibiotics and the overuse of the existing ones, represents one of the major threats to public health, being responsible for treatment failure and limiting the therapeutic options [14,15]. Considering the difficulties and the long-duration required for obtaining new synthetic antibiotics, the rapidity of gaining resistance to the existing antimicrobials and the toxicity of some classes of antibiotics, the exploration of the antimicrobial potential of the natural compounds, including natural polymers, raised a particular interest, sustaining the large amount of researches in this field [16]. In addition to this, natural compounds play a key role as sources of new scaffolds for antibiotics; according to Newman and Crag reports, in the last 40 years, there were introduced 162 molecules as antibacterial agents, from which only 36 molecules were completely synthetic, over 55% of them including natural products and semi-synthetic derivatives [8,17]. 

Chitosan is one of the natural polymers which raised a strong interest for researchers, in the Scopus database being available up to 17,000 citations, reflecting a particular concern in terms of chemistry and application of chitosan [18]. Possessing some exceptional properties as biodegradability, biocompatibility, nontoxicity, non-antigenicity, low-cost and numerous pharmacological properties as antimicrobial, antitumor, antioxidant, antidiabetic, immunoenhancing, chitosan has found wide applications in medicine, pharmacy, food industry and textiles industry [9,19]. 

This review approaches the physico-chemical and pharmacological properties of chitosan and its derivatives, being focused on the antimicrobial potential including mechanism of action, factors that influence the antimicrobial activity and the activity against resistant strains, topics of great interest in the context of the concern raised by the available therapeutic options of infections, especially with resistant strains. 

## 2. Sources, Preparation Methods and Physico-Chemical Properties of Chitosan and Its Derivatives 

**Figure 1:**
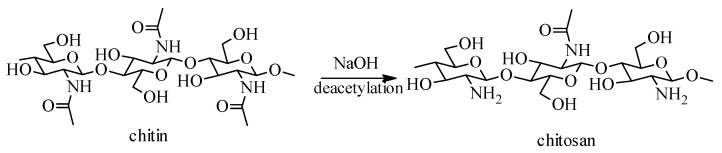
Scheme for chitosan production from chitin.

Chitosan is a linear cationic polysaccharide derived from chitin (extracted from the exoskeleton of crustaceans) by a deacetylation process, which has been identified in insects, but also in fungi and algae [20]. If cellulose is the substance that gives tissue resistance and hardness in the plant world, among the lower marine animals, this role is played by chitosan. The difference between cellulose and chitosan consists in the fact that the 2-hydroxyl group of the cellulose structure has been replaced, in the case of chitosan, with the acetamido group (Figure 1) [21]. 

Chitosan, poly-α(1,4)-2-amino-2-deoxy-β-D-glucan is a natural, hydrophilic, nontoxic, biocompatible and biodegradable polysaccharide, usually obtained by *N*-deacetylation of α-chitin, using alkaline solutions (40–50%), at 100–160 °C, for several hours (Figure 1) [22].

One of the most important chemical characteristics of chitosan is the degree of deacetylation, which can influence its use in many applications. In addition, the degree of deacetylation expresses the number of free amino groups, thus making the difference between chitin and chitosan. Based on this idea, chitin with a degree of deacetylation of at least 75% is known as chitosan. The deacetylation process involves the removal of acetyl functional groups from the chitin molecule chain with the release of amino groups [23]. The ratio of acetylated and deacetylated glucosamine residues plays an important role in the balance between hydrophilic and hydrophobic interactions [24].

From chemical point of view, chitosan is a polycationic polymer consisting of glucosamine-linked *N*-acetyl-glucosamine units at the 1-4 position containing free amino and hydroxyl groups, which makes it susceptible to a series of structural modulations [21]. These structural modulations have led to the obtaining of some derivatives with different biomedical applications. 

Chitosan can be easily modified by physical and chemical reactions in order to obtain derivatives with different structures, properties and applications. In general, the chemical changes made on chitosan structure refer to three reactive sites: the amino group at C_2_ and the -OH groups from C_3_ and C_6_ [25,26,27]. The functional amino groups in the chitosan structure provide the possibility of performing acetylation, quaternization, condensation reactions with aldehydes and ketones (to form Schiff bases), alkylation etc. So far, chemical changes have been made to chitosan to produce chitosan sulfate, chitosan trimethylate, chitosan thiolate, hydroxyalkyl chitosan, carboxyalkyl chitosan, and phosphorylated chitosan. The purpose of such chemical reactions is to obtain new derivatives which possess antibacterial, antifungal, antiviral, antacid, anti-ulcer, nontoxic, non-allergic properties, etc. [28]. Although chitosan is considered a nontoxic polymer, the structural modulations implemented on it may turn it into a more or less toxic compound, so any unreacted reagent must be carefully removed. 

### 2.1. Chitosan Sulfate

Chitosan sulfate, known for its in vitro anticoagulant activity, was obtained by a three-step synthesis process [29]:

- Obtaining the quaternary ammonium salt of chitosan. To an aqueous solution of *N*-(3-chloro-2-hydroxypropyl)-trimethylammonium chloride, 15% NaOH was added, to pH 8. The resulting mixture was stirred at room temperature for 48 h and another 24 h at 50 °C to form the quaternary ammonium salt of chitosan.
- Obtaining the sulphating agent N(SO_3_Na)_3_. An aqueous solution of sodium nitrite was continuously stirred over a solution of sodium bisulfite, at 90 °C, over 90 min.
- Obtaining the quaternary ammonium salt of chitosan sulfate. The quaternary ammonium salt of chitosan was added over the solution containing the sulfating agent under mechanical agitation, at room temperature, then the reaction product obtained was concentrated and dried at 40 °C.

Other synthetic methods of some chitosan sulfate derivatives are cited in the literature [30,31].

### 2.2. Chitosan Trimethylate

**Figure 2:**
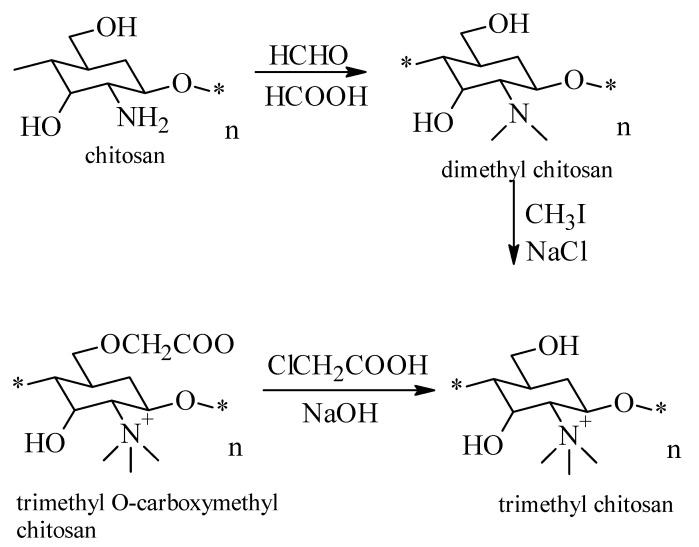
Scheme for the preparation of the trimethylated derivative of chitosan. “*” means that this units binds to other units through glycosidic bonds.

The synthesis process of the trimethylated derivative of chitosan is carried out in three steps (Figure 2) [32,33,34], and aims to improve physico-chemical and biological properties of chitosan:

- obtaining the dimethyl chitosan by reaction of formic acid with formaldehyde at a temperature of 70 °C for 118 h;
- the reaction of dimethyl chitosan with iodoform under continuous stirring at a temperature of 70 °C, to obtain trimethyl chitosan;
- the last step is the reaction of trimethyl chitosan with monocloracetic acid in alkaline medium.

### 2.3. Chitosan Thiolate

**Figure 3:**
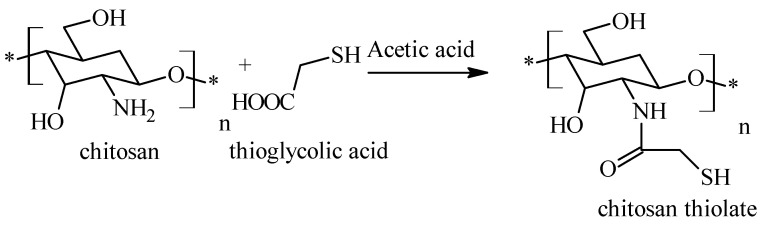
Scheme for the preparation of chitosan thiolate.

**Figure 4:**
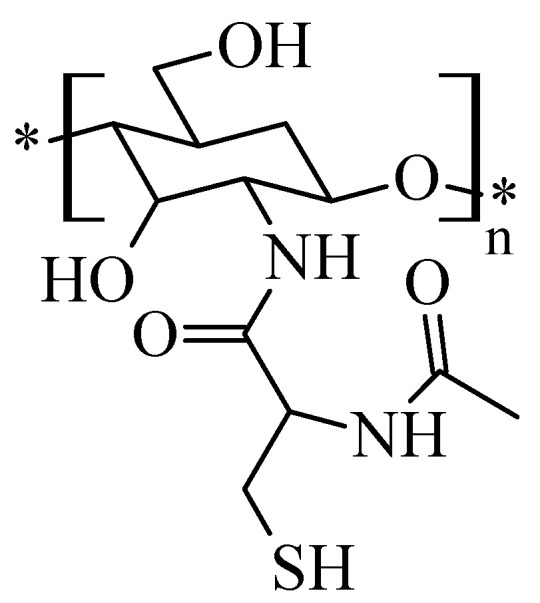
Structure of the chitosan-*N*-acetylcysteine derivative.

Thiol chitosan is obtained by treating pure chitosan with thioglycolic acid in acetic acid medium (Figure 3). The reaction took place at a pH of 5, for avoid the formation of disulfide bonds (once the coupling reaction is performed), the reaction being mediated by a carbodiimide. At the end, the mixture was incubated in dark, at room temperature, for about 4 h, and then, the reaction product was isolated by dialysis of the polymer solution [35]:

Similarly-structured compounds were obtained by treating chitosan with *N*-acetylcysteine, involving the participation of the amino group of chitosan and the carboxyl group of *N*-acetylcysteine (Figure 4) [36]:

Such derivatives have often been cited in the literature as compounds with improved solubility to chitosan and with particular emphasis on their interactions with human conjunctival epithelial cells [37]. 

### 2.4. Chitosan-N-hydroxy-propyl Derivatives

**Figure 5:**
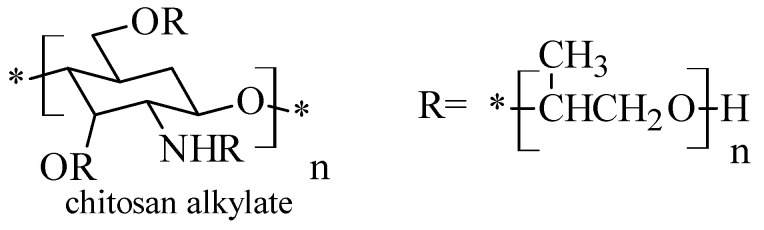
Hydroxypropyl derivatives of chitosan.

A series of hydroxypropyl derivatives of chitosan (Figure 5), which have been studied for in vitro antimicrobial activity, are known to have different degrees of substitution [38]. The technique for their preparation involves, in a first step, shaking chitosan with aqueous NaOH at room temperature for 2 h and then maintaining the mixture at −18 °C for 10 days. Subsequently, isopropyl alcohol and propylene oxide were added over this mixture to afford the corresponding hydroxypropyl derivatives.

### 2.5. Chitosan N-Alkyl Derivatives

**Figure 6:**
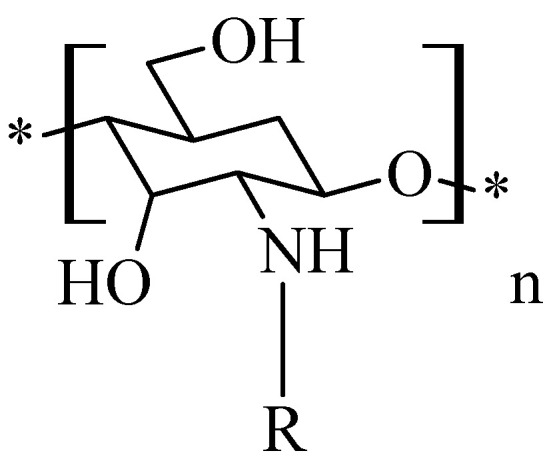
Structure of *N*-alkylated chitosan derivatives.

The *N*-alkylated derivatives of chitosan (Figure 6) are obtained by substitution of the primary amino group with various mono or disaccharides such as lactose, maltose, cellobiose [39]. NaCNBH3 was used and after that the mixture was kept at room temperature for about 48 h, until the white product was formed. At the end, the reaction product was washed with methanol and dried. The changes made resulted in the production of chitosan derivatives with increased solubility and important antimicrobial properties.

### 2.6. Chitosan N-Carboxymethyl Derivatives

**Figure 7:**
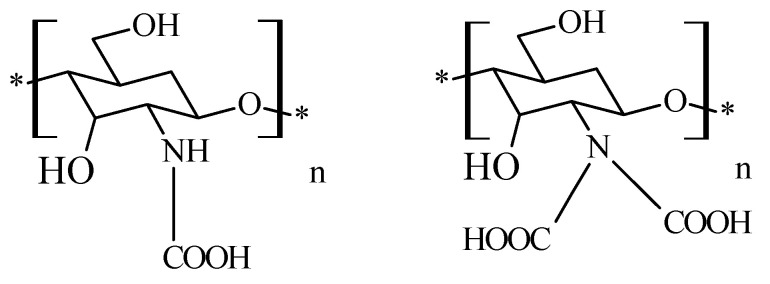
Structure of chitosan mono and dicarboxymethylates derivatives.

The principle of producing *N*,*N*-carboxymethylated chitosan derivatives (Figure 7) involves the reaction between the primary amine group of chitosan and the carbonyl group of glyoxylic acid, followed by hydrogenation with NaBH_4_ or NaCNBH_3_ to form *N*-monocarboximethyl-chitosan or *N*,*N*-dicarboxymethyl-chitosan; the substitution on the -NH_2_ group being dependent on the ratio of the reagents. Fully substituted *N*,*N′* -dicarboxymethyl-chitosan and its di-sodium salt are suitable for promotion of the osteogenis [40].

### 2.7. Phosphorylated Chitosan

**Figure 8:**
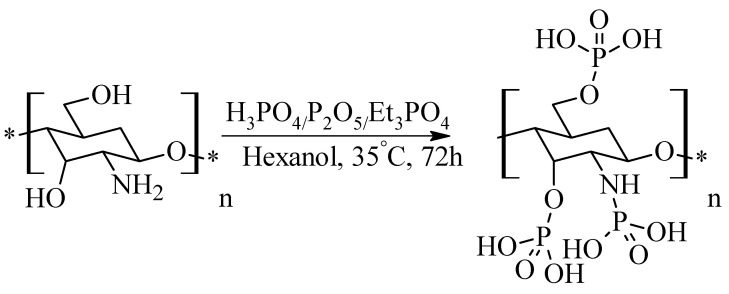
Synthesis method of phosphorylated chitosan.

Synthesis of novel phosphorylated chitosan derivatives consists of treating chitosan with a mixture of P_2_O_5_, H_3_PO_4_ and Et_3_PO_4_ in hexanol and stirring it, for 72 h at 35 °C (Figure 8) [41]. This type of derivatives have important orthopedic and tissue engineering applications. 

Another method for obtaining phosphorylated chitosan is described in the literature and is based on the treatment of chitosan with urea in dimethylformamide medium and the subsequent addition of orthophosphoric acid [42]. The reaction mixture thus obtained is kept at 150 °C for one hour.

## 3. Pharmacological Potential of Chitosan and Its Derivatives. Applications in Current Pharmacotherapy 

The first known use of chitosan was in the form of flexible and durable films applied to Stradivarius violins, with the role of increasing resistance to degradation over time [43]. In the early 1960s, chitosan was extensively studied for its ability to fix red blood cells, being designed and studied as a hemostatic agent. Today, chitosan is known to properties such as the ability to bind water, fat, bioactivity, biodegradability, nontoxicity, biocompatibility, ability to accelerate wound healing and antimicrobial activity [44]. Due to these characteristics, chitosan has been assigned a number of applications, whether used as such or in combination with other natural polymers in the food industry, the pharmaceutical industry, the textile industry, agriculture, the cosmetics industry [45].

### 3.1. Antimicrobial Activity

#### 3.1.1. Antibacterial Activity

Bacterial infections, both community and hospital-acquired, represent a major cause of mortality, morbidity and prolonged hospitalization, closing a vicious circle and leading to an unfavorable outcome for patients and to an economic burden [15,46,47]. The treatment of bacterial infections, especially of those hospital-acquired, raise serious concerns related to the rapid development of antibiotic resistance phenomenon and rapid spread of multidrug resistant strains, leading to a drastic limitation of the therapeutic options [48]. 

The bacterial species of great interest at this moment are those having the ability to gain resistance through different mechanisms of genetic transfer such as transformation, conjugation, transduction and mobile genetic elements [49]. The species of particular interest include *Staphylococcus aureus*, *Enterococcus* spp., *Enterobacteriaceae* family, *Pseudomonas aeruginosa* and *Acinetobacter* spp. [47,50,51]. 

Taking into consideration these aspects, there is an increased pressure to develop new antibacterial agents, active against resistant strains or to valorize the antimicrobial activity of natural compounds [52,53]. 

Chitosan is a natural polymer with a broad antibacterial spectrum [54], including Gram-positive and Gram-negative bacterial strains. Chitosan proved to be active against *Staphylococcus aureus*, *Staphylococcus epidermidis*, *Bacillus cereus*, *Bacillus megaterium*, *Lysteria monocytogenes*, *Lactobacillus brevis*, *Escherichia coli*, *Pseudomonas aeruginosa*, *Pseudomonas fluorescens*, *Salmonella typhimurium* [9]. 

Chitosan’s antimicrobial activity against a variety of bacteria is well known but is also limited by low solubility [55] which is explicable by its rigid crystalline structure [56]. However, the presence of free amino groups offers the possibility of derivatization of chitosan by various controlled chemical reactions, thus obtaining more soluble compounds. 

Changes made to its structure, such as quaternization and hydrophilic substitution, increase solubility. Chitosan guanidilate derivatives, chitosan galactosylate etc. have been included in a series of studies that have demonstrated antimicrobial activity against various bacteria such as *Staphylococcus aureus*, *Bacillus subtilis*, *Escherichia coli*, *Pseudomonas aeruginosa*. All these derivatives exhibit a much higher activity than unchanged chitosan at pH 7 [39]. 

##### Mechanism of Action 

The mechanism of antibacterial activity of chitosan and its derivatives was intensively studied and discussed; the interaction between chitosan and microorganism appears to be a complex phenomenon that is not fully understood [57]. The mechanism was supposed to be different between Gram-positive and Gram-negative bacteria because of different cell surface characteristics [52]. Gram-negative bacteria seems to be more susceptible to chitosan and its derivatives due to a higher negative charge of the cell surface [45,57,58]. 

The key role in the antibacterial activity of chitosan and its derivatives appears to be the electrostatic interactions between the polycationic structure of chitosan and the anionic groups found on the bacterial cell surface, leading to the alteration of the cell wall (Gram-positive) or outer membrane (Gram-negative), followed by disturbances in cytoplasmic membrane permeability and, consequently, the loss of essential constituents as enzymes, nucleotides, ions, and death of the bacterial cell [54,57,59,60]. 

Other modes of antibacterial action, relying on the molecular weight and the physical state, were proposed. Low molecular weight chitosan, both water soluble and chitosan-based small nanoparticles could pass through cell wall and inhibit mRNA synthesis and DNA transcription [45,52]. By contrast, high molecular weight chitosan and larger size nanoparticles could form an impermeable layer at the cell surface, affecting the transport of vital constituents into the bacterial cell [57,61]. Another proposed mechanism was related to the ability of chitosan to chelate metal cations and essential nutrients for bacterial cell growth [52]. 

##### Factors That Influence the Antibacterial Activity 

**Figure 9:**
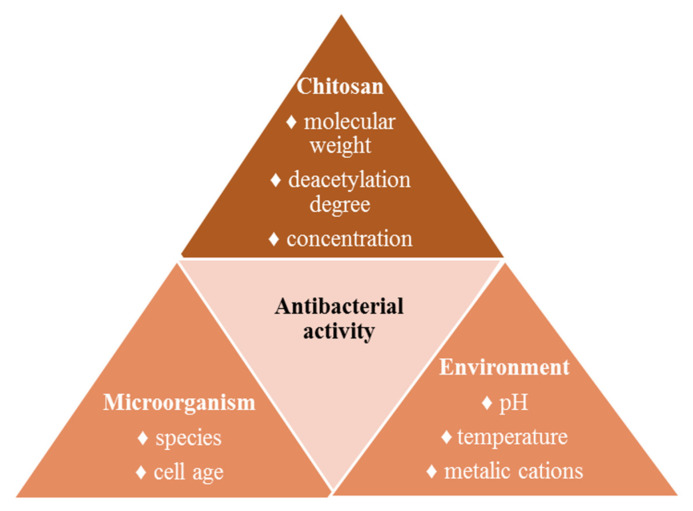
Factors that influence the antibacterial activity of chitosan.

The studies carried out during the last two decades evidenced that the antibacterial activity of chitosan and its derivatives depends on several factors related to chitosan (molecular weight, deacetylation degree, concentration), microorganism (species, cell age) and environmental factors (pH, presence of metal cations, temperature) (Figure 9) [45,54]. The most relevant factors influencing the antibacterial activity will be discussed below. 

Molecular weight: The influence of the molecular weight of chitosan on the antibacterial activity was studied using *S. aureus* and *E.coli* strains. The results highlighted that for Gram-positive bacteria, the antimicrobial activity increases with the increase of the molecular weight, while for Gram-negative bacteria, the antibacterial activity of chitosan is stronger with decrease in molecular weight [9]. However, this conclusion should be investigated for other Gram-positive and Gram-negative bacterial strains. In this regard, Ortega-Ortiz et al. studied the influence of the molecular weight of chitosan on the antimicrobial activity against *P. aeruginosa* and *P. oleovorans* strains. The results evidenced that the low molecular weight chitosan showed an inhibiting percent of 72.52%, compared with 64.57% recorded for high molecular weight chitosan [62]. Concerning resistant strains, chitosan with low molecular weight proved to have a stronger antibacterial activity [63,64].

Deacetylation degree: Deacetylation degree, directly influencing the positive charge density, has a significant influence on the antibacterial activity of chitosan and its derivatives; it was supposed that higher positive charge density determines stronger electrostatic interactions [45]. Some studies support that a high deacetylation degree, enhancing the positive charge density, was associated with a superior antibacterial activity against *S. aureus* [65,66].

pH: The antibacterial activity of chitosan depends on pH, chitosan being soluble in acidic medium and becoming cationic at pH values below the molecules pKa [67]. Chitosan proved stronger antibacterial activity at pH values lower than 6.5 [57], the inhibitory effect decreasing when increasing pH [65]. Contrary, Yang et al. reported *N*-alkylated chitosan derivatives with increased antibacterial activity against *E. coli* when increasing pH values between 5.0 and 7.5 [39]. These controversial results support that the mechanism of antibacterial activity is more complex and is not dependent exclusively on the positive charge of the amino groups [45]. 

##### Antibacterial Activity against Resistant Strains

Given the global threat represented by the antimicrobial resistance, the development of new antimicrobial agents active against multidrug resistant pathogens, is of particular interest [64]. The most problematic bacterial species at this moment are *S. aureus* (MRSA—methicillin resistant *Staphylococcus aureus*, VRSA—vancomycin resistant *Staphylococcus aureus*), *S. epidermidis* (MRSE—methicillin resistant *Staphylococcus epidermidis*), *Enterococcus* spp. (VRE—vancomycin resistant *Enterococcus*), *E. coli*, *K. pneumoniae*, *P. aeruginosa*, *Acinetobacter* spp.

The studies concerning the antimicrobial activity against resistant strains were focused on three main directions: the antibacterial activity of chitosan with different physicochemical characteristics against Gram-positive and Gram-negative resistant strains, the antimicrobial activity of chitosan-based polymeric systems as nanofibers/nanoparticles against resistant strains and the synergism between chitosan and other antimicrobial agents. 

Concerning the first direction, Park et al. investigated the antibacterial activity of low molecular weight chitosan against drug-resistant *S. aureus* and *P. aeruginosa*, clinical isolates. The results showed that the antibacterial activity of chitosan was more effective against all drug-resistant *P. aeruginosa* isolates and against some *S. aureus* isolates than against ATCC strains [64]. In addition to this, the antibacterial activity of chitosan was studied in vivo using mice infected with drug resistant *P. aeruginosa* and *S. aureus*, the results revealing a good survival rate and reduced bacterial colonization [64]. 

Supotngarmkul et al. investigated the antibacterial activity of chitosan against *E. faecalis*, ATCC strains and clinical isolates, the results revealing a good antimicrobial activity. However, the susceptibility profile of the clinical isolates was not mentioned [68]. 

Costa et al. compared the antibiofilm activity of chitosan against MRSA and MSSA (methicillin sensible *Staphylococcus aureus*) using reference strains and clinical multiresistant isolates. The results showed methicillin resistance did not affect chitosan’s activity; moreover, the higher susceptibility was registered for MRSA and for low molecular weight chitosan [63]. These results are strongly encouraging, given the clinical impact and concerns related to the treatment of MRSA infections. 

Infections with *Acinetobacter* spp. represent a serious threat, due to its remarkable ability to acquire resistance and to the rapid spread of resistant strains in the hospital environment [69]. Saito et al. obtained lysozyme–chitosan oligosaccharides conjugates with good antibacterial activity against *P. aeruginosa*, *A. baumannii* and MRSA [69]. Further studies using multidrug resistant clinical isolates of *A. baumannii* would be of great interest. 

Concerning the second direction, Abadi et al. evaluated, for the first time, the antibacterial activity of chitosan nanofibers against *Clostridium difficile*, ATCC strains and clinical isolates possessing resistance genes, showing similar results. These first results are promising, taking into account the problems encountered in treating *Clostridium difficile* infections [70]. 

Zhang et al. prepared chitosan nanoparticles loaded with essential oils with significant antibacterial activity against multidrug resistant *K. pneumoniae*. The study showed the ability of chitosan with low molecular weight and a deacetylation degree of 75% to enhance the antibacterial activity of essential oils [71]. 

Regarding the third direction, the synergistic action of cephalosporin drugs and chitosan was proved by Jamil et al. who obtained chitosan-cefotaxime nanoparticles with broad-spectrum antibiofilm and antibacterial activity against clinical isolates of multi-drug-resistant *K. pneumoniae*, *E. coli*, *P. aeruginosa* and MRSA [72]. Approaching a similar direction, Oliveria et al. obtained chitosan low molecular weight-based nanoparticles containing ceftriaxone and a natural extract. The antibacterial activity of these polymeric systems was evaluated against multi-drug-resistant *Enterobacteriaceae*, namely ESBL-producing (extended-spectrum beta-lactamases) *E. coli* clinical isolates and carbapenemase-producing *K. pneumoniae* clinical isolates. The results revealed a considerable improvement in the antibacterial activity, the polymeric systems reaching an improvement of 133 times in the minimum inhibitory activity of ceftriaxone [73]. 

According to these studies, the results concerning the activity of chitosan and its derivatives against resistant strains, both per se and in different chitosan-antibiotic nanoparticles, are promising and encouraging. Given the rapid acquirement of antibiotic resistance, the development of new chitosan-antibiotic formulations active against resistant strains is of great interest. 

#### 3.1.2. Antiviral Activity

During the last decades, HIV infection, hepatitis B and C, were among the most threatening viral infections, determining chronic diseases difficult to be controlled and associated with a high mortality and morbidity [74]. Nowadays, other viruses are overwhelming the medical community, such as SARS-Cov-2, influenza viruses [75]. 

The antiviral activity of chitosan and its derivatives was less studied and less explored, probably because of difficulties and special requirement in cultivating viruses. However, the literature data provides some information concerning the antiviral activity of chitosan.

Artan et al. evidenced a chitosan-oligosaccharide (3–5 kDa) that at nontoxic concentrations inhibited HIV-1 replication through inhibiting HIV-1-induced syncytia formation and reducing the production on p24 antigen. Moreover, this oligosaccharide blocked viral entry and virus-cell fusion, presenting the potential of a novel candidate for the development of new anti-HIV agents [76]. In addition to this, the anti-HIV activity of a chitosan-conjugate and the corresponding nanoparticles was studied using MT4 cell line; the results highlighted that chitosan-conjugate nanoparticles may be used as a targeting and sustained polymeric prodrugs, improving treatment efficacy and decreasing side effects in antiretroviral treatment [9]. 

Loutfy et al. prepared low molecular weight chitosan-based nanoparticles loaded with curcumin designed to increase the antiviral activity of curcumin. The nanoparticles were evaluated against hepatitis C virus genotype 4a using human hepatoma cells, the results proving 100% inhibition of viral entry and replication [74]. 

Zheng et al. reported, using a mouse model, that the intranasal administration of chitosan is efficient in preventing influenza A H7N9 infection, the mechanism proposed being related to the stimulation of innate immune system [77]. 

Recently, Sharma et al. speculated the potential of using chitosan as a potential molecule against SARS-Cov-2 virus, the mechanism proposed being related to targeting CD 147 receptors, a novel route for invasion of the virus into cells [78,79].

Most studies reveal the capacity of chitosan to act as a drug carrier, being used for obtaining delivery systems that could improve the therapeutic effect of other antiviral agents as foscarnet [80], acyclovir [81], ribavirin [82], amantadine [83]. 

#### 3.1.3. Antifungal Activity

Fungal infections, with an increasing incidence worldwide [84] represent a serious concern especially in immunocompromised patients, including the following categories: diabetes, HIV infection, different neoplasms, immunosuppressive therapy and prolonged hospitalization in intensive care units [85,86,87]. Whether it occurs as primary infection or a superinfection after a viral or bacterial infectious disease, fungal infections raise problems related to the treatment options and the outcome is frequently unfavorable [88,89]. 

The fungal species most frequently involved in human pathology belong to the Genus *Candida*, *Aspergillus*, *Cryptococcus* and *Pneumocystis*, being responsible for more than 90% of reported deaths due to fungal infections [88]. 

The problems concerning the treatment of fungal infections are related to few therapeutic options available and to the relatively high toxicity of the existing drugs [89,90]. In addition to this, the resistance phenomenon to antifungal agents is more and more common and drastically limits the use of the available drugs [91,92]. 

Considering these aspects, the development of new antifungal agents, with a more favorable safety profile, is of significant interest [93,94]. 

Chitosan proved important antifungal activity, even if the mechanisms of action are less studied that those involved in the antibacterial activity. Based on the available data, fungi appear to be more sensitive to antimicrobial activity of chitosan than bacteria [45]. The antifungal activity of chitosan depends on several factors as molecular weight, deacetylation degree, chitosan concentration and pH [92]. The influence of the molecular weight and deacetylation degree seems to be dependent on tested strains [92]. Concerning chitosan concentration, values between 1% and 5% proved to ensure an optimal antifungal activity [95]. Similar to antibacterial activity, the antifungal activity of chitosan is greater at lower pH values [45]. 

Concerning the mechanisms involved in the antifungal activity, the first studies highlighted as the main effect the permeabilization of the yeast cells and the disruption of the cell membrane by chitosan and its derivatives [96]. According to Pena et al., chitosan exerts some important effects against *Candida* spp. which may be responsible for its antifungal activity: a large efflux of K^+^ and increase of Ca^2+^ uptake and the inhibition of respiration, fermentation and cell viability [96]. 

*Candida* spp. were the most investigated pathogenic yeast concerning the antifungal activity of chitosan, being the most frequent etiological agents isolated from fungal infections. Alburquenque et al. studied in vitro antifungal activity of low molecular weight chitosan against 105 clinical *Candida* isolates, including fluconazole-resistant strains. The results proved a significant antifungal activity, chitosan inhibiting over 89.9% of the tested strains. In addition to this, for nine of the tested strains, chitosan proved minimum inhibitory concentrations lower than fluconazole [95]. 

The antifungal activity of chitosan when associated with antifungal drugs used in clinical practice received significant attention, but the results were contradictory. Low molecular weight chitosan with a molecular weight of 70 kDa and a deacetylation degree over 75% proved promising anti-*Candida* activity, whereas its combination with fluconazole did not have a synergistic effect [95]. Ganan et al. reported a chitosan-oligosaccharide with a molecular weight of 15 kD showing great synergistic effects against *Candida* spp. when associated with different antifungals as fluconazole, voriconazole, miconazole and amphotericin B [97]. Lo et al. studied the antifungal activity of chitosan with different molecular weights and deacetylation degrees against drug resistant *Candida* strains, as well as synergistic effects in combination with fluconazole, amphotericin B and caspofungin. The results revealed a remarkable synergistic effect for the association chitosan-fluconazole, the combination with other antifungal agents being indifferent. Moreover, the combination of chitosan and fluconazole proved to be effective against drug-resistant strains. In addition to this, the study highlights the influence of the molecular weight and deacetylation degree of chitosan on the antifungal activity [92]. 

### 3.2. Other Pharmacological Effects

Being included in various pharmaceutical forms (powders, gels), chitosan is used in tissue regeneration, its biocompatibility being important in reducing local inflammation. One of the most important uses of chitosan is the treatment of wounds caused by burns, which has a remarkable compatibility with tissue regeneration [98]. It is also used successfully in anti-tumor medication as well as in reducing serum cholesterol levels [99]. The latter property was explained by a series of mechanisms including electrostatic interaction between lipids and aminopolysaccharides, which leads to inhibition of lipid absorption, as well as increased bile acid excretion, which leads to increased amounts of fats eliminated by feces [100].

#### 3.2.1. Healing Activity

The loss of skin integrity, caused by trauma, burns, chronic conditions, etc. has become a critical issue in medical practice. The tissue regeneration process is complex, with a number of growth factors, DNA, RNA, being involved in mediating cellular response and modulating cellular behavior [101]. Wound healing can be compromised or delayed by many local and systemic factors, leading to the need for specialized treatment [102]. Chitosan accelerates the healing process by stimulating fibroblast growth while affecting macrophage activity. In this context, the substituents obtained by the derivatization of chitosan have particularly attracted attention through their properties, such as biocompatibility, hemostatic activity, antimicrobial properties, and the ability to accelerate the healing process of wounds. In the form of sponges, chitosan can be used as a scarring agent in severe burns, trauma, venous ulceration, etc. [103]. In addition, the scarring process can be improved by incorporating antibacterial agents such as ciprofloxacin, norfloxacin, sulfonamides [104] into the sponge structure. Together with sponges, hydrogels, films, and chitosan-based fibers can also be used in the tissue regeneration process [105]. All of these systems have to fulfill a number of properties such as biocompatibility, favorable mechanical properties, appropriate degradation rate, ability to preserve metabolic functions, and the creation of a suitable environment providing hydration, oxygen permeability, water vapor, carbon dioxide, release of bioactive compounds and act as a barrier for microorganisms [106].

#### 3.2.2. Hemostatic Effect

The hemostatic effect, manifested by blood clotting, is an important step in the healing process of the wounds. It has been demonstrated that chitin derivatives exhibit this effect due to their physical and chemical properties, particularly due to the presence of primary amino groups, and exhibit the anticoagulation property of the blood [107].

#### 3.2.3. Antioxidant Activity

Antioxidants play an important role in protecting the human body against damage caused by reactive oxygen species. It is known that reactive oxygen species are unfavorable to the wound healing process due to the harmful effects on the cells and tissues. It has been reported that the topical use of compounds with antioxidant properties is beneficial in the wound healing process and that it provides the protection of tissues against oxidative processes [108]. The antioxidant properties of chitin and its derivatives against free radicals such as 1,1-diphenyl-picryl-hydrazyl (DPPH), hydroxyl, superoxide and peroxide have been extensively studied over time. This has been shown to be dependent on the degree of deacetylation and the concentration of the polymer. Primary amino groups in the chitosan structure play an important role by interacting with the free radicals, forming NH^3+^ groups [109].

Among the four forms of amino groups: primary amino groups, imino groups, secondary amino groups and quaternized amino groups, the latter have demonstrated an impressive antioxidant activity towards the hydroxyl radicals. Particularly deacetylated low molecular weight chitosan has antioxidant properties and can be considered a natural antioxidant [110].

### 3.3. Drug Delivery Systems

Chitin and its derivatives can also be used as controlled release drug delivery systems. It is especially important for such a system to be removed from the body after drug delivery and, in particular, not to be toxic [111]. Chitin derivatives, such as *N*-succinyl-chitosan, carboxymethyl-chitin, hydroxyethyl-chitin, are used extensively in pharmaceutical technology [112]. Such systems could be achieved by the use of chitosan through different encapsulation methods and can be used to encapsulate different active principles: proteins/peptides, growth factors, anti-inflammatory compounds, antibiotics, compounds with anti-tumor action [113]. 

## 4. Conclusions and Perspectives

This review approaches one of the most widely used biodegradable polymers, namely chitosan, targeting its obtainment and its derivatives synthesis, their pharmacological properties, focusing on the antimicrobial activity, as well as possible applications in current pharmacotherapy. Given the concerns raised by the treatment of infectious diseases, especially the development of antibiotic resistance and the rapid spread of resistant strains, the antimicrobial potential of natural compounds is of great interest. Chitosan possesses some unique properties as biocompatibility, biodegradability, lack of toxicity, accessibility, along with the possibility to be structurally modulated at the free amino and hydroxyl groups that provides the opportunity to improve some physico-chemical and pharmacological properties. The most studied chitosan derivatives were chitosan sulfate, chitosan trimethylate, chitosan thiolate, chitosan-*N*-acetylcysteine derivative, *N*-alkylated chitosan derivatives, chitosan *N*-carboxymethyl derivatives, phosphorylated chitosan. The antibacterial activity of chitosan and its derivatives against the resistant strains of particular concern (*S. aureus*, *E. coli*, *P. aeruginosa*, *Enterococcus* spp., *K. pneumoniae*, *A. baumannii*) presents all the theoretical premises to be valorized in therapy. In addition to this, studies regarding the synergistic effect against resistant strains when associating some consecrated antibiotics with chitosan, together with the possibility to include these antibiotics in chitosan-based delivery systems, proved encouraging results and opened new perspectives in overcoming the problems raised by antibiotic resistance. In relation to this, future research should be directed to the development of different types of chitosan-based formulations (nanofibers, nanoparticles, hydrogels) including as target drug antibiotics susceptible to the development of resistance phenomenon. Moreover, these new formulations could associate different antimicrobial agents obtained from natural sources in order to enhance the antibacterial activity of the antibiotic. Not at least, considering the property of chitosan to ensure a sustained release of the drugs encapsulated in its matrix, these formulations could improve the pharmacokinetic profile of some antibiotics. Even less studied, the direct antiviral and antifungal activity of chitosan, coupled with the possibility to develop chitosan-based drug delivery systems, is also promising. Not at least, chitosan and its derivatives present other pharmacological effects such as wound healing, hemostatic and antioxidant activity, properties that are beneficial for the evolution of some infections, especially those of the skin and soft tissues. Due to their remarkable activities, we can consider these compounds as new therapeutic approaches for the treatment and prevention of various types of infections. Corroborating these ideas, pharmaceutical formulations based on chitosan and its derivatives present all the premises for a multi-target therapy.

## Data Availability

Not applicable.

## References

[1] Sonia T.A., Sharma C.P. (2014). 6–Polymers in Oral Insulin Delivery. Oral Delivery of Insulin.

[2] Fonte P., Araújo F., Silva C., Pereira C., Reis S., Santos H.A., Sarmento B. (2015). Polymer-based nanoparticles for oral insulin delivery: Revisited approaches. Biotechnol. Adv..

[3] Elsabee M.Z., Abdou E.S. (2013). Chitosan based edible films and coatings: A review. Mat. Sci. Eng. C Mater..

[4] Agrawal U., Sharma R., Gupta M., Vyas S.P. (2014). Is nanotechnology a boon for oral drug delivery?. Drug Discov. Today.

[5] Thakur S., Verma A., Sharma B., Chaudhary J., Tamulevicius S., Thakur V.K. (2018). Recent developments in recycling of polystyrene based plastics. Curr. Opin. Green Sustain. Chem..

[6] Song R., Murphy M., Li C., Ting K., Soo C., Zheng Z. (2018). Current development of biodegradable polymeric materials for biomedical applications. Drug Des. Dev. Ther..

[7] Reddy M.S.B., Ponnamma D., Choudhary R., Sadasivuni K.K. (2021). A comparative review of natural and synthetic biopolymer composite scaffolds. Polymers.

[8] Mattio L.M., Catinella G., Dallavalle S., Pinto A. (2020). Stilbenoids: A natural arsenal against bacterial pathogens. Antibiotics.

[9] Ngo D.-H., Vo T.-S., Ngo D.-N., Kang K.-H., Je J.-Y., Pham H.N.-D., Byun H.-G., Kim S.-K. (2015). Biological effects of chitosan and its derivatives. Food Hydrocolloid.

[10] Bernkop-Schnürch A., Dünnhaupt S. (2012). Chitosan-based drug delivery systems. Eur. J. Pharm. Biopharm..

[11] Lee K.Y., Mooney D.J. (2012). Alginate: Properties and biomedical applications. Prog. Polym. Sci..

[12] Frieri M., Kumar K., Boutin A. (2017). Antibiotic resistance. J. Infect. Public Health.

[13] Sharland M., Saroey P., Berezin E.N. (2015). The global threat of antimicrobial resistance—The need for standardized surveillance tools to define burden and develop interventions. J. Pediatr..

[14] Bertesteanu S., Chifiriuc M.C., Grumezescu A.M., Printza A.G., Marie-Paule T., Grumezescu V., Mihaela V., Lazar V., Grigore R. (2014). Biomedical applications of synthetic, biodegradable polymers for the development of anti-infective strategies. Curr. Med. Chem..

[15] O’Rourke A., Beyhan S., Choi Y., Morales P., Chan A.P., Espinoza J.L., Dupont C.L., Meyer K.J., Spoering A., Lewis K. (2020). Mechanism-of-action classification of antibiotics by global transcriptome profiling. Antimicrob. Agents Chemother..

[16] Kamaruzzaman N.F., Tan L.P., Hamdan R.H., Choong S.S., Wong W.K., Gibson A.J., Chivu A., Pina M.F. (2019). Antimicrobial polymers: The potential replacement of existing antibiotics?. Int. J. Mol. Sci..

[17] Newman D.J., Cragg G.M. (2020). Natural products as sources of new drugs over the nearly four decades from 01/1981 to 09/2019. J. Nat. Prod..

[18] Abd El-Hack M.E., El-Saadony M.T., Shafi M.E., Zabermawi N.M., Arif M., Batiha G.E., Khafaga A.F., Abd El-Hakim Y.M., Al-Sagheer A.A. (2020). Antimicrobial and antioxidant properties of chitosan and its derivatives and their applications: A review. Int. J. Biol. Macromol..

[19] Shariatinia Z. (2019). Pharmaceutical applications of chitosan. Adv. Colloid Interface.

[20] Meramo-Hurtado S., Alarćon-Suesca C., González-Delgado A.D. (2020). Exergetic sensibility analysis and environmental evaluation of chitosan production from shrimp exoskeleton in Colombia. J. Clean. Prod..

[21] Batista M.K.S., Pinto L.F., Gomes C.A.R., Gomes P. (2006). Novel highly-soluble peptide-chitosan polymers: Chemical synthesis and spectral characterization. Carbohyd. Polym..

[22] Carraher C.E., Seymour R.B. (2008). Polymer Chemistry.

[23] Khan T.A., Peh K.K., Ch’ng H.S. (2002). Reporting degree of deacetylation values of chitosan: The influence of analytical methods. J. Pharm. Pharm. Sci..

[24] Ladet S., David L., Domard A. (2008). Multi-membrane hydrogels. Nature.

[25] Zhang F., Wang B., Jie P., Zhu J., Cheng F. (2021). Preparation of chitosan/lignosulfonate for effectively removing Pb(II) in water. Polymer.

[26] Zhang Y., Zhao M., Cheng Q., Wang C., Li H., Han X., Fan Z., Su G., Pan D., Li Z. (2021). Research progress of adsorption and removal of heavy metals by chitosan and its derivatives: A review. Chemosphere.

[27] Tan W., Zhang J., Mi Y., Dong F., Li Q., Guo Z. (2020). Enhanced antifungal activity of novel cationic chitosan derivative bearing triphenylphosphonium salt via azide-alkyne click reaction. Int. J. Biol. Macromol..

[28] Pillai C.K.S., Paul W., Sharma C.P. (2009). Chitin and chitosan polymers: Chemistry, solubility and fiber formation. Prog. Polym. Sci..

[29] Fan L., Wu P., Zhang J., Gao S., Wang L., Li M., Sha M., Xie W., Nie M. (2012). Synthesis and anticoagulant activity of the quaternary ammonium chitosan sulfates. Int. J. Biol. Macromol..

[30] Zhong Z., Ji X., Xing R., Liu S., Guo Z., Chen X., Li P. (2007). The preparation and antioxidant activity of the sulfanilamide derivatives of chitosan and chitosan sulfates. Bioorg. Med. Chem..

[31] Wang T., Zhou Y., Xie W., Chen L., Zheng H., Fan L. (2012). Preparation and anticoagulant activity of *N*-succinyl chitosan sulfates. Int. J. Biol. Macromol..

[32] Xu T., Xin M., Li M., Huang H., Zhou S. (2010). Synthesis, characteristic and antibacterial activity of *N*,*N*,*N*-trimethyl chitosan and its carboxymethyl derivatives. Carbohyd. Polym..

[33] Benediktsdóttir B.E., Gaware V.S., Rúnarsson Ö.V., Jόnsdόttir S., Jensen K.J., Másson M. (2011). Synthesis of *N*,*N*,*N*-trimethyl chitosan homopolymer and highly substituted *N*-alkyl-*N*,*N*-dimethyl chitosan derivatives with the aid of di-*tert*-butyldimethylsilyl chitosan. Carbohyd. Polym..

[34] Geisberger G., Gyenge E.B., Maake C., Patzke G.T. (2013). Trimethyl and carboxymethyl chitosan carriers for bio-active polymer–inorganic nanocomposites. Carbohyd. Polym..

[35] Anitha A., Deepa N., Chennazhi K.P., Nair S.V., Tamura H., Jayakumar R. (2011). Development of mucoadhesive thiolated chitosan nanoparticles for biomedical applications. Carbohyd. Polym..

[36] Hombach J., Hoyer H., Bernkop-Schnürch A. (2008). Thiolated chitosans: Development and in vitro evaluation of an oral tobramycin sulphate delivery system. Eur. J. Pharm. Sci..

[37] Schuerer N., Stein E., Inic-Kanada A., Ghasemian E., Stojanovic M., Montanaro J., Bintner N., Hohenadl C., Sachsenhofer R., Barisani-Asenbauer T. (2018). Effects of chitosan and chitosan *N*-acetylcysteine solutions on conjunctival epithelial cells. J. EuCornea.

[38] Peng Y., Han B., Liu W., Xu X. (2005). Preparation and antimicrobial activity of hydroxypropyl chitosan. Carbohyd. Res..

[39] Yang T.-C., Chou C.-C., Li C.-F. (2005). Antibacterial activity of *N*-alkylated disaccharide chitosan derivatives. Int. J. Food Microbiol..

[40] An N.T., Dung P.L., Thien D.T., Dong N.T., Nhi T.T.Y. (2008). An improved method for synthesizing N,N′-dicarboxymethylchitosan. Carbohyd. Polym..

[41] Jayakumar R., Nagahama H., Furuike T., Tamura H. (2008). Synthesis of phosphorylated chitosan by novel method and its characterization. Int. J. Biol. Macromol..

[42] Li B., Huang L., Wang X., Ma J., Xie F. (2011). Biodegradation and compressive strength of phosphorylated chitosan/chitosan/ hydroxyapatite bio-composites. Mater. Des..

[43] Lang G., Zakaria M.B., Wan Muda W.M., Abdullah M.P. (1995). Chitosan Derivatives—Preparation and Potential Uses. Chitin and Chitosan: The Versatile Environmentally Friendly Modern Materials.

[44] Yang J.M., Yang S.J., Lin H.T., Wu T.-H., Chen H.-J. (2008). Chitosan containing PU/Poly(NIPAAm) thermosensitive membrane for wound dressing. Mat. Sci. Eng. C Mater..

[45] Kong M., Chen X.G., Xing K., Park H.J. (2010). Antimicrobial properties of chitosan and mode of action: A state of the art review. Int. J. Food Microbiol..

[46] Kaprou G.D., Bergšpica I., Alexa E.A., Alvarez-Ordóñez A., Prieto M. (2021). Rapid methods for antimicrobial resistance diagnostics. Antibiotics.

[47] Bassetti M., Carnelutti A., Peghin M. (2017). Patient specific risk stratification for antimicrobial resistance and possible treatment strategies in gram-negative bacterial infections. Expert Rev. Anti-Inf..

[48] Guitor A.K., Raphenya A.R., Klunk J., Kuch M., Alcock B., Surette M.G., McArthur A.G., Poinar H.N., Wright G.D. (2020). Capturing the resistome: A targeted capture method to reveal antibiotic resistance determinants in metagenomes. Antimicrob. Agents Chemother..

[49] Almasaudi S.B. (2018). Acinetobacter spp. as nosocomial pathogens: Epidemiology and resistance features. Saudi J. Biol. Sci..

[50] Agodi A., Barchitta M., Quattrocchi A., Maugeri A., Aldisio E., Marchese A.E., Mattaliano A.R., Tsakris A. (2015). Antibiotic trends of *Klebsiella pneumoniae* and *Acinetobacter baumannii* resistance indicators in an intensive care unit of Southern Italy, 2008–2013. Antimicrob. Resist. Infect. Control.

[51] Garnacho-Montero J., Dimopoulos G., Poulakou G., Akova M., Cisneros J.M., de Waele J., Petrosillo N., Seifert H., Timsit J.F., Vila J. (2015). Task force on managament and prevention of *Acinetobacter baumannii* infections in the ICU. Intens. Care Med..

[52] Matica M.A., Aachmann F.L., Tøndervik A., Sletta H., Ostafe V. (2019). Chitosan as a wound dressing starting material: Antimicrobial properties and mode of action. Int. J. Mol. Sci..

[53] Mantravadi P.K., Kalesh K.A., Dobson R.C.J., Hudson A.O., Parthasarathy A. (2019). The quest for novel antimicrobial compounds: Emerging trends in research, development, and technologies. Antibiotics.

[54] Zou P., Yang X., Wang J., Li Y., Yu H., Zhang Y., Liu G. (2016). Advances in characterisation and biological activities of chitosan and chitosan oligosaccharides. Food Chem..

[55] Xie Y., Liu X., Chen Q. (2007). Synthesis and characterization of water-soluble chitosan derivate and its antibacterial activity. Carbohyd. Polym..

[56] Ma G., Yang D., Zhou Y., Xiao M., Kennedy J.F., Nie J. (2008). Preparation and characterization of water-soluble *N*-alkylated chitosan. Carbohyd. Polym..

[57] Younes I., Rinaudo M. (2015). Chitin and chitosan preparation from marine sources. Structure, properties and applications. Mar. Drugs..

[58] Chung Y.C., Su Y.P., Chen C.C., Jia G., Wang H.L., Wu J.C.G., Lin J.G. (2004). Relationship between antibacterial activity of chitosans and surface characteristics of cell wall. Acta Pharmacol. Sin..

[59] Raafat D., Bargen K.V., Haas A., Sahl H.-G. (2008). Insights into the mode of action of chitosan as an antibacterial compound. Appl. Environ. Microb..

[60] Chung Y.-C., Chen C.-Y. (2008). Antibacterial characteristics and activity of acid-soluble chitosan. Bioresour. Technol..

[61] Eaton P., Fernandes J.C., Pereira E., Pintado M.E., Malcata F.X. (2008). Atomic force microscopy study of the antibacterial effects of chitosans on *Escherichia coli* and *Staphylococcus aureus*. Ultramicroscopy.

[62] Ortega-Ortiz H., Gutierrez-Rodriguez B., Cadenas-Pliego G., Ibarra-Jimenez L. (2010). Antibacterial Activity of Chitosan and the Interpolyelectrolyte Complexes of Poly(acrylic acid)-Chitosan. Braz. Arch. Biol. Technol..

[63] Costa E.M., Silva S., Tavaria F.K., Pintado M.M. (2016). Insights into chitosan antibiofilm activity against methicillin-resistant *Staphylococcus aureus*. J. Appl. Microbiol..

[64] Park S.-C., Nam J.-P., Kim J.-H., Kim Y.-M., Nah J.-W., Jang M.-K. (2015). Antimicrobial action of water-soluble β-chitosan against clinical multi-drug resistant bacteria. Int. J. Mol. Sci..

[65] Kong M., Chen X.G., Liu C.S., Liu C.G., Meng X.H., Yu L.J. (2008). Antibacterial mechanism of chitosan microspheres in a solid dispersing system against E. coli. Colloid. Surf. B Biointerfaces.

[66] Takahashi T., Imai M., Suzuki I., Sawai J. (2008). Growth inhibitory effect on bacteria of chitosan membranes regulated by the deacetylation degree. Biochem. Eng. J..

[67] Lim S.-H., Hudson S.M. (2004). Synthesis and antimicrobial activity of a water-soluble chitosan derivative with a fiber-reactive group. Carbohyd. Res..

[68] Supotngarmkul A., Panichuttra A., Ratisoontorn C., Nawachinda M., Matangkasombut O. (2020). Antibacterial property of chitosan against *E. faecalis* standard strain and clinical isolates. Dent. Mater. J..

[69] Saito H., Sakakibara Y., Sakata A., Kurashige R., Murakami D., Kageshima H., Saito A., Miyazaki Y. (2019). Antibacterial activity of lysozyme-chitosan oligosaccharide conjugates (LYZOX) against *Pseudomonas aeruginosa*, *Acinetobacter baumannii* and methicillin-resistant *Staphylococcus aureus*. PLoS ONE.

[70] Shahini Shams Abadi M., Mirzaei E., Bazargani A., Gholipour A., Heidari H., Hadi N. (2020). Antibacterial activity and mechanism of action of chitosan nanofibers against toxigenic *Clostridioides (Clostridium) difficile* isolates. Ann. Ig..

[71] Zhang F., Ramachandran G., Mothana R.A., Noman O.M., Alobaid W.A., Rajivgandhi G., Manoharan N. (2020). Anti-bacterial activity of chitosan loaded plant essential oil against multi drug resistant *K. pneumoniae*. Saudi J. Biol. Sci..

[72] Jamil B., Habib H., Abbasi S.A., Ihsan A., Nasir H., Imran M. (2016). Development of cefotaxime impregnated chitosan as nano-antibiotics: De novo strategy to combat biofilm forming multi-drug resistant pathogens. Front. Microbiol..

[73] De Oliveira M.S., Oshiro-Junior J.A., Sato M.R., Conceição M.M., Medeiros A.C.D. (2020). Polymeric nanoparticle associated with ceftriaxone and extract of Schinopsis Brasiliensis Engler against *Multiresistant Enterobacteria*. Pharmaceutics.

[74] Loutfy S.A., Elberry M.H., Farroh K.Y., Mohamed H.T., Mohamed A.A., Mohamed E.B., Faraag A.H.I., Mousa S.A. (2020). Antiviral activity of chitosan nanoparticles encapsulating curcumin against hepatitis C virus genotype 4a in human hepatoma cell lines. Int. J. Nanomed..

[75] Meng Q., Sun Y., Cong H., Hu H., Xu F.-J. (2021). An overview of chitosan and its application in infectious diseases. Drug Deliv. Transl. Res..

[76] Artan M., Karadeniz F., Karagozlu M.Z., Kim M.-M., Kim S.-K. (2010). Anti-HIV-1 activity of low molecular weight sulfated chitooligosaccharides. Carbohyd. Res..

[77] Zheng M., Qu D., Wang H., Sun Z., Liu X., Chen J., Li C., Li X., Chen Z. (2016). Intranasal administration of chitosan against Influenza A (H7N9) virus infection in a mouse model. Sci. Rep..

[78] Sharma N., Modak C., Singh P.K., Kumar R., Khatri D., Singh S.B. (2021). Underscoring the immense potential of chitosan in fighting a wide spectrum of viruses: A plausible molecule against SARS-CoV-2?. Int. J. Biol. Macromol..

[79] Safarzadeh M., Sadeghi S., Azizi M., Rastegari-Pouyani M., Pouriran R., Hoseini M.H.M. (2021). Chitin and chitosan as tools to combat COVID-19: A triple approach. Int J Biol Macromol..

[80] Russo E., Gaglianone N., Baldassari S., Parodi B., Cafaggi S., Zibana C., Donalisio M., Cagno V., Lembo D., Caviglioli G. (2014). Preparation, characterization and in vitro antiviral activity evaluation of foscarnet-chitosan nanoparticles. Colloid. Surface B.

[81] Kubbinga M., Nguyen M.A., Staubach P., Teerenstra S., Langguth P. (2015). The influence of chitosan on the oral bioavailability of acyclovir—a comparative bioavailability study in humans. Pharm. Res..

[82] Giuliani A., Balducci A.G., Zironi E., Colombo G., Bortolotti F., Lorenzini L., Galligioni V., Pagliuca G., Scagliarini A., Calzà L. (2018). In vivo nose-to-brain delivery of the hydrophilic antiviral ribavirin by microparticle agglomerates. Drug Deliv..

[83] Shital L., Bowen J., Badhan R. (2016). Development and evaluation of a novel intranasal spray for the delivery of amantadine. J. Pharm. Sci..

[84] Lockhart S.R., Guarner J. (2019). Emerging and reemerging fungal infections. Semin. Diagn. Pathol..

[85] Pagano L., Akova M., Dimopoulos G., Herbrecht R., Drgona L., Blijlevens N. (2011). Risk assessment and prognostic factors for mould-related diseases in immunocompromised patients. J. Antimicrob. Chemoth..

[86] Garbee D.D., Pierce S.S., Manning J. (2017). Opportunistic fungal infections in critical care units. Crit. Care Nurs. Clin..

[87] Silva R.F.e. (2010). Fungal infections in immunocompromised patients. J. Bras. Pneumol..

[88] Schmiedel Y., Zimmerli S. (2016). Common invasive fungal diseases: An overview of invasive candidiasis, aspergillosis, cryptococcosis, and *Pneumocystis* pneumonia. Swiss Med. Wkly..

[89] Paramythiotou E., Frantzeskaki F., Flevari A., Armaganidis A., Dimopoulos G. (2014). Invasive fungal infections in the ICU: How to approach, how to treat. Molecules.

[90] Perfect J.R. (2017). The antifungal pipeline: A reality check. Nat. Rev. Drug. Discov..

[91] Jensen R.H. (2016). Resistance in human pathogenic yeasts and filamentous fungi: Prevalence, underlying molecular mechanism and link to the use of antifungals in humans and the environment. Dan. Med. J..

[92] Lo W.-H., Deng F.-S., Chang C.-J., Lin C.-H. (2020). Synergistic antifungal activity of chitosan with fluconazole against *Candida albicans*, *Candida tropicalis*, and fluconazole-resistant strains. Molecules.

[93] Parente-Rocha J.A., Bailão A.M., Amaral A.C., Taborda C.P., Paccez J.D., Borges C.L., Pereira M. (2017). Antifungal resistance, metabolic routes as drug targets, and new antifungal agents: An overview about endemic dimorphic fungi. Mediat. Inflamm..

[94] Fuentefria A.M., Pippi B., Dalla Lana D.F., Donato K.K., Andrade S.F. (2017). Antifungals discovery: An insight into new strategies to combat antifungal resistance. Lett. Appl. Microbiol..

[95] Alburquenque C., Bucarey S.A., Andrónico N.C., Urzúa B., Hermosilla G., Tapia C.V. (2010). Antifungal activity of low molecular weight chitosan against clinical isolates of *Candida* spp.. Med. Mycol..

[96] Peña A., Sánchez N.S., Calahorra M. (2013). Effects of chitosan on *Candida albicans*: Conditions for its antifungal activity. BioMed Res. Int..

[97] Ganan M., Lorentzen S.B., Aam B.B., Eijsink V.G.H., Gaustad P., Sørlie M. (2019). Antibiotic saving effect of combination therapy through synergistic interactions between well-characterized chito-oligosaccharides and commercial antifungals against medically relevant yeasts. PLoS ONE.

[98] Jiang Z., Zhang K., Du L., Cheng Z., Zhang T., Ding J., Li W., Xu B., Zhu M. (2021). Construction of chitosan scaffolds with controllable microchannel for tissue engineering and regenerative medicine. Mater. Sci. Eng. C.

[99] Dash M., Chiellini F., Ottenbrite R.M., Chiellini E. (2011). Chitosan—A versatile semi-synthetic polymer in biomedical applications. Prog. Polym. Sci..

[100] Gallaher C.M., Munion J., Hesslink R., Wise J., Gallaher D.D. (2000). Cholesterol reduction by glucomannan and chitosan is mediated by changes in cholesterol absorption and bile acid and fat excretion in rats. J. Nutr..

[101] Ye J., Xie C., Wang C., Huang J., Yin Z., Heng B.C., Chen X., Shen W. (2021). Promoting musculoskeletal system soft tissue regeneration by biomaterial-mediated modulation of macrophage polarization. Bioact. Mater..

[102] Dias A.M.A., Rey-Rico A., Oliveira R.A., Marceneiro S., Alvarez-Lorenzo C., Concheiro A., Júnior R.N.C., Braga M.E.M., de Sousa H.C. (2013). Wound dressings loaded with an anti-inflammatory jucá (*Libidibia ferrea*) extract using supercritical carbon dioxide technology. J. Supercrit. Fluids.

[103] Dragostin O.M., Samal S.K., Dash M., Lupascu F., Panzariu A., Tuchilus C., Ghetu N., Danciu M., Dubruel P., Pieptu D. (2016). New antimicrobial chitosan derivatives for wound dressing applications. Carbohydr. Polym..

[104] Öztürk E., Agalar C., Keçeci K., Denkbas E.B. (2006). Preparation and characterization of ciprofloxacin-loaded alginate/chitosan sponge as wound dressing material. J. Appl. Polym. Sci..

[105] Dragostin O.M., Samal S.K., Lupascu F., Panzariu A., Dubruel P., Lupascu D., Tuchilus C., Vasile C., Profire L. (2015). Development and Characterization of Novel Films Based on Sulfonamide-Chitosan Derivatives for Potential Wound Dressing. Int. J. Mol. Sci..

[106] Liu X., Ma L., Mao Z., Gao C., Jayakumar R., Prabaharan M., Muzzarelli R.A.A. (2011). Chitosan-Based Biomaterials for Tissue Repair And Regeneration. Chitosan for Biomaterials II.

[107] Okamoto Y., Yano R., Miyatake K., Tomohiro I., Shigemasa Y., Minami S. (2003). Effects of chitin and chitosan on blood coagulation. Carbohyd. Polym..

[108] Gouthamchandra K., Mahmood R., Manjunatha H. (2010). Free radical scavenging, antioxidant enzymes and wound healing activities of leaves extracts from *Clerodendrum infortunatum* L.. Environ. Toxicol. Pharmacol..

[109] Xie W., Xu P., Liu Q. (2001). Antioxidant activity of water-soluble chitosan derivatives. Bioorg. Med. Chem. Lett..

[110] Vinsova J., Vavrikova E. (2008). Recent advances in drugs and prodrugs design of chitosan. Curr. Pharm. Des..

[111] Tao F., Ma S., Tao H., Jin L., Luo Y., Zheng J., Xiang W., Deng H. (2021). Chitosan-based drug delivery systems: From synthesis strategy to osteomyelitis treatment—A review. Carbohyd. Polym..

[112] Dev A., Mohan J.C., Sreeja V., Tamura H., Patzke G.R., Hussain F., Weyeneth S., Nair S.V., Jayakumar R. (2010). Novel carboxymethyl chitin nanoparticles for cancer drug delivery applications. Carbohyd. Polym..

[113] Lupascu F., Dash M., Samal S.K., Dubruel P., Lupusoru C.E., Lupusoru R.V., Dragostin O., Profire L. (2015). Development, optimization and biological evaluation of chitosan scaffold formulations of new xanthine derivatives for treatment of type-2 diabetes mellitus. Eur. J. Pharm. Sci..

